# Phylogenetic and phenotypic evidence of *Bannoa* (Erythrobasidiales, Erythrobasidiaceae) revealing one new genus and four new species in China

**DOI:** 10.3897/mycokeys.130.183686

**Published:** 2026-03-20

**Authors:** Zhi-Wen Xi, Bing-Yan Song, Chun-Yue Chai, Qiu-Hong Niu, Feng-Li Hui

**Affiliations:** 1 School of Life Science, Nanyang Normal University, Nanyang 473061, China Research Center of Henan Provincial Agricultural Biomass Resource Engineering and Technology, Nanyang Normal University Nanyang China; 2 Research Center of Henan Provincial Agricultural Biomass Resource Engineering and Technology, Nanyang Normal University, Nanyang 473061, China School of Life Science, Nanyang Normal University Nanyang China

**Keywords:** Basidiomycota, blastoconidia-forming yeasts, multigene analysis, plant leaves

## Abstract

*Bannoa*, a genus within the family Erythrobasidiaceae of the order Erythrobasidiales, consists of blastoconidia-forming yeasts that are widely distributed across the globe. Members of this genus are known to form associations with various plant leaves. Currently, 17 species are described and recognized as valid members of the genus, although the full extent of its diversity and global distribution remains insufficiently explored. In the present study, yeast strains were isolated from the surface of diverse plant leaves collected in the Guizhou and Henan Provinces of China, and were subsequently identified using a combination of molecular and phenotypic techniques. Phylogenetic analysis, based on concatenated SSU, ITS, LSU, and *TEF1-α* sequences, along with phenotypic characterization, revealed the establishment of a new monotypic genus, *Lumyongozyma*, as well as the description of four new species: *Bannoa dicranopteri***sp. nov**., *B. neixiangensis***sp. nov**., *B. pini***sp. nov**., and *Lumyongozyma fatsiae***sp. nov**. This study highlights the novel geographical distribution and significant species diversity of *Bannoa* in China, contributing valuable data for ongoing research in fungal systematics and evolution.

## Introduction

The genus *Bannoa*, a group of basidiomycetous yeasts within the family Erythrobasidiaceae, was first introduced by Hamamoto et al. (2002) and later emended by Wang et al. (2015a) based on multi-gene phylogenetic analysis. Currently, *Bannoa* includes 17 recognized species, three of which—*Sporobolomyces bischofiae*, *S. ogasawarensis*, and *S. syzygii*—were formerly classified within the *Bannoa* clade (Hamamoto et al. 2002, 2011; Nagahama et al. 2006; Hamamoto 2011; Wang et al. 2015b). Members of this genus share several distinctive phenotypic characteristics, such as the formation of orange to salmon-red colonies, the production of ballistoconidia, polar budding, an inability to form pseudohyphae, the synthesis of coenzyme Q-10(H2), and a non-fermentative metabolic nature (Hamamoto 2011; Wang et al. 2015a). Additionally, two teleomorphic species—*B. hahajimensis* and *B. tropicalis*—are known to produce unicellular basidia either laterally on a clamp connection or terminally at the hyphae (Hamamoto et al. 2002; Hamamoto 2011; Parra and Aime 2019).

Most species of *Bannoa* have been reported to be isolated from dead, asymptomatic, or infected leaves and are considered important phyllosphere-inhabiting yeasts (Hamamoto et al. 2002; Parra and Aime 2019; Tan et al. 2021; Chai et al. 2023; Krwanji 2023). Among them, *B. bischofiae*, *B. ellipsoidea*, *B. guamensis*, *B. tropicalis*, *B. hahajimensis*, *B. ogasawarensis*, and *B. syzygii* were isolated from dead and infected leaves, indicating they are pathogenic (Hamamoto et al. 2002; Parra and Aime 2019; Chai et al. 2023). The remaining species were obtained from asymptomatic leaves, suggesting they are epiphytic. Additionally, *B. tropicalis*, *B. guamensis*, and several environmental isolates have been found in association with rust sori, where they may coinhabit this microniche during their yeast stage (Parra and Aime 2019).

The genus *Bannoa* is widely distributed geographically; however, its species diversity remains poorly understood. In China, 11 species have been reported, including eight species—*B. ellipsoidea*, *B. foliicola*, *B. hyperici*, *B. lonicerae*, *B. pseudofoliicola, B. pseudoscolopiae, B. quercus*, and *B. scolopiae*—that were originally described in the country (Zang et al. 1998; Li et al. 2020; Chai et al. 2023; Sun et al. 2025). Current members of the genus have been documented in specific regions, including *B. syzygii* from Beijing City (Li et al. 2020), *B. hahajimensis* from Fujian Province (Li et al. 2020), *B. hyperici*, *B. lonicerae*, *B. pseudoscolopiae, B. quercus*, and *B. scolopiae* from Guizhou province (Sun et al. 2025), *B. ogasawarensis* from Hebei Province (Li et al. 2020), *B. ellipsoidea*, *B. foliicola*, and *B. pseudofoliicola* from Henan Province (Chai et al. 2023), and *B. hahajimensis* and *B. ogasawarensis* from Zhejiang Province (Zang et al. 1998).

China, located in the northern hemisphere, offers a temperate environment conducive to supporting *Bannoa* species. To fully uncover the genus’s diversity and enhance our understanding of its ecological and evolutionary significance, comprehensive surveys across diverse ecosystems and substrates, particularly in under-sampled regions, are essential. During a survey of the diversity of phyllosphere-inhabiting yeasts in Guizhou and Henan Provinces, China, we encountered ten yeast strains that could not be identified as any known species. Our aim in this study is to clarify their molecular phylogenetic positions and to delimit them based on phenotypic data and molecular evidence.

## Materials and methods

### Sample collection and yeast isolation

Semi-withered leaves were collected from the Guizhou and Henan Provinces of China. The leaf samples were stored in sterile flasks and transported under refrigerated conditions within 24 hours of collection. Yeast strains were isolated from the surface of the plant leaves using the ballistospore-fall method, as previously described (Nakase and Takashima 1993; Chai et al. 2023). The leaves were cut into small pieces and affixed to the inner lid of a Petri dish with a thin layer of petroleum jelly. The Petri dish contained yeast extract-malt extract (YM) agar medium (0.3% yeast extract, 0.3% malt extract, 0.5% peptone, 1% glucose, and 2% agar), supplemented with 0.01% chloramphenicol to inhibit bacterial growth. The plates were incubated at 20 °C and monitored daily for colony development. Distinct yeast morphotypes were picked and purified by streaking onto fresh YM agar plates. All purified yeast strains were suspended in YM broth supplemented with 20% (v/v) glycerol and stored at –80 °C.

### Phenotypic examination

Morphological, physiological, and biochemical characterizations were performed following the standard methods outlined by Kurtzman et al. (2011). Colony morphology was observed on YM agar after 7 days of incubation at 20 °C. Cellular characteristics were observed in YM broth after 3 days of incubation at 20 °C. Mycelium formation was examined by culturing on corn meal agar (CMA; 2% cornmeal infusion and 2% agar) in slide culture at 20 °C for two weeks. The ballistoconidium-forming activity of all new species was assessed using the inverted-plate method (do Carmo-Sousa and Phaff 1962) with CMA at 17 °C. The potential sexual states of the new species were investigated for individual strains and strain pairs on CMA, potato dextrose agar (PDA; 20% potato infusion, 2% glucose, and 1.5% agar), and yeast carbon base plus 0.01% ammonium sulfate (YCBS) agar at 20 °C for two months (Hamamoto et al. 2002; Parra and Aime 2019). Glucose fermentation was tested in a liquid medium using Durham fermentation tubes. Carbon and nitrogen source assimilation tests were conducted in liquid medium, with starved inocula used for the nitrogen assimilation tests (Kurtzman et al. 2011). Growth at various temperatures (15, 20, 25, 30, 35, and 37 °C) was determined by cultivation on YM agar. The novel taxonomic descriptions and proposed names were deposited in MycoBank (http://www.mycobank.org; 20 November 2025).

### DNA extraction and molecular sequencing

Genomic DNA was extracted from the yeast strains using the Ezup Column Yeast Genomic DNA Purification Kit, following the manufacturer’s protocol (Sangon Biotech, China). Four gene loci were sequenced, including the small ribosomal subunit (SSU) rRNA gene, the internal transcribed spacer (ITS) region, the D1/D2 domain of the large subunit (LSU) rRNA gene, and the translation elongation factor 1-α (*TEF1-α*) gene. The primer pairs NS1/NS8 (White et al. 1990), ITS1/ITS4 (White et al. 1990), NL1/NL4 (Kurtzman and Robnett 1998), and EF1-526F/EF1-1567R (Rehner and Buckley 2005) were used to amplify the SSU, ITS, LSU, and *TEF1-α* regions, respectively.

PCR amplification was performed as described by Toome et al. (2013) for the SSU, ITS, and LSU regions. For the *TEF1-α* gene, a touchdown PCR protocol was used as outlined by Wang et al. (2014). Purified products were sent to Sangon Biotech Inc. (Shanghai, China) for Sanger sequencing, using the same primers on an automated ABI 3730 XL capillary sequencer. Forward and reverse sequence reads were assembled into contigs using BioEdit v.7.1.3.0 software (Hall 1999). The newly obtained sequences were subsequently submitted to GenBank (https://www.ncbi.nlm.nih.gov/genbank/).

### Phylogenetic analyses

BLAST searches of the newly generated sequences indicated that the investigated yeasts belong to the family Erythrobasidiaceae. To examine their phylogenetic relationships, the sequences generated in this study, along with those of all formally described species in Erythrobasidiaceae from previous work and deposited in the NCBI GenBank database (Table 1), were used for phylogenetic analysis. The DNA sequences of each locus (SSU, ITS, LSU, and *TEF1-α*) were aligned separately using MAFFT v.7.110 (Katoh and Standley 2013) and then manually adjusted to remove ambiguous regions in BioEdit v.7.1.3.0 (Hall 1999). Alignments of the different loci were concatenated into a supermatrix using PhyloSuite v.1.2.3 (Zhang et al. 2020). Maximum Likelihood (ML) and Bayesian Inference (BI) analyses were performed using RAxML v.8.2.3 (Stamatakis 2014) and MrBayes v.3.2.7a (Ronquist et al. 2012), respectively. In the ML analysis, the GTRGAMMA model of evolution was applied, and bootstrap (BS) values were assessed through 1,000 rapid bootstrap replicates. For BI analysis, six Markov Chain Monte Carlo (MCMC) chains were run simultaneously for 50 million generations under the best evolutionary models selected using MrModeltest 2.3 (Posada and Crandall 1998), with trees sampled every 1,000 generations. The first 25% of the generated trees were discarded as burn-in, and the remaining trees were used to estimate Bayesian posterior probabilities (BPPs) for the clades. The consensus tree was visualized with BS values and BPPs in Figtree v.1.4.3 (Andrew 2016) and edited in Inkscape v.1.1.

**Table 1.** Sequences used for phylogenetic analysis. Entries in bold were newly generated in this study.

Species	Strains no.	Locality	GenBank accession no.			
			SSU	ITS	LSU D1/D2	TEF1-α
*Bannoa banksiae*	BRIP 71965a^T^	Australia	–	OR491787	–	–
*B. bischofiae*	JCM 10338^T^	Japan	AB035721	AB035721	NG_058609	AB127094
*B. dicranopteri*	NYUN 24379^T^	China	PX551769	PP837685	PP849685	PX505481
*B. dicranopteri*	NYNU 24344	China	PX551768	PP837684	PP837681	PX505487
*B. dicranopteri*	NYNU 24348	China	PX551770	–	PP837687	PX505482
*B. ellipsoidea*	NYNU 2110396^T^	China	OP221010	OM014197	OM014195	OP725922
foliicola	NYNU 208237^T^	China	OP218261	MW365541	MW365544	OP750517
*B. guamensis*	CBS 16127^T^	USA	MK254996	MK287350	MK255006	MK491345
*B. hahajimensis*	JCM 10336^T^	Japan	AB035897	AB035897	NG_042311	KJ707750
*B. hyperici*	CGMCC 2.6710^T^	China	–	OL853642	OL853642	OL952729
*B. lonicerae*	CGMCC 2.6600^T^	China	–	OL853650	OL853650	OL952728
*B. macarangae*	BRIP 28272^T^	Australia	–	NR_175756	NG_079569	–
*B. neixiangensis*	NYNU 2211164^T^	China	PX551774	OP954739	OP954732	PX505483
*B. neixiangensis*	NYNU 243106	China	PX551775	PX551776	PX551777	PX505486
*B. ogasawarensis*	JCM 10330^T^	Japan	AB035717	AB035717	NG_058699	AB127095
*B. pini*	NYNU 232195^T^	China	PX551771	OQ851908	OQ851907	PX505484
*B. pini*	NYNU 232234	China	PX551772	OQ851909	OQ857289	–
*B. pini*	NYNU 232242	China	PX551773	OQ857287	OQ857288	PX505485
*B. pseudoscolopiae*	CGMCC 2.6716^T^	China	–	OL853662	OL853662	OL952724
*B. quercus*	CGMCC 2.6722^T^	China	–	OL853682	OL853682	OL952725
*B. rosea*	CBS 16128^T^	USA	–	MK287351	MK255007	MK491346
*B. scolopiae*	CGMCC 2.6717^T^	China	–	OL853646	OL853646	OL952732
*B. syzygii*	JCM 10337^T^	Japan	AB035720	AB035720	NG_058700	AB127096
*B. tropicalis*	CBS 16087^T^	USA	MK255003	MK287360	MK255016	MK491353
*B. pseudofoliicola*	NYNU 2110469^T^	China	OP221018	OM014200	OM014198	OP750518
*Cyrenella elegans*	CBS 274.8^T^	USA	NG_061174	NR_145383	NG_058875	KJ707830
*Erythrobasidium elongatum*	CBS 8080^T^	Australia	NG_063449	NR_073306	NG_059254	AB127099
*E. hasegawianum*	JCM 1545^T^	USA	D12803	NR_111008	AF131058	KJ707776
*E. leptospermi*	BRIP 66853^T^	Australia	–	NR_175759	NG_079571	–
*E. nanyangense*	NYNU 208200^T^	China	OP218268	MW362360	MW362359	OP313688
*E. primogenitum*	BRIP 72389e^T^	Australia	–	NR_182613	OP598058	–
*E. proteacearum*	BRIP 66871^T^	Australia	–	NR_175760	NG_079572	–
*E. turpiniae*	NYNU 2110435^T^	China	OP218271	OM014199	OM014196	OR785452
*E. yunnanense*	CBS 8906^T^	China	NG_063520	NR_155098	NG_059190	AB127100
*Hasegawazyma lactosa*	CBS 5826^T^	Japan	D45366	NR_073295	NG_057668	AB127098
*Lumyongozyma fatsiae*	NYNU 22915^T^	China	PX444847	OP740374	OP740373	PX505479
*Lumyongozyma fatsiae*	NYNU 241191	China	PX551767	PX551843	PX551778	PX505480
*Naohidea sebacea*	CBS 8477^T^	–	KP216515	NR_121324	NG_042442	KJ707783

Note. ^T,^ type strain.

## Results

### Molecular phylogeny

Sequences from 33 strains were used for phylogenetic analysis, with *Naohidea sebacea* CBS 8477 designated as the outgroup (Li et al. 2020; Chai et al. 2023). The total length of the concatenated dataset for the four regions across the 33 strains was 3663 bp, comprising 1749 bp for SSU, 589 bp for ITS, 569 bp for LSU, and 756 bp for *TEF1-α*. Phylogenetic analysis of the combined SSU, ITS, LSU, and *TEF1-α* dataset strongly supported Erythrobasidiaceae as an independent family (BS = 100%, BPPs = 1.0). Within Erythrobasidiaceae, three new lineages emerged from *Bannoa*, and one new independent clade clustered with *Bannoa*, receiving full support.

**Figure 1.**
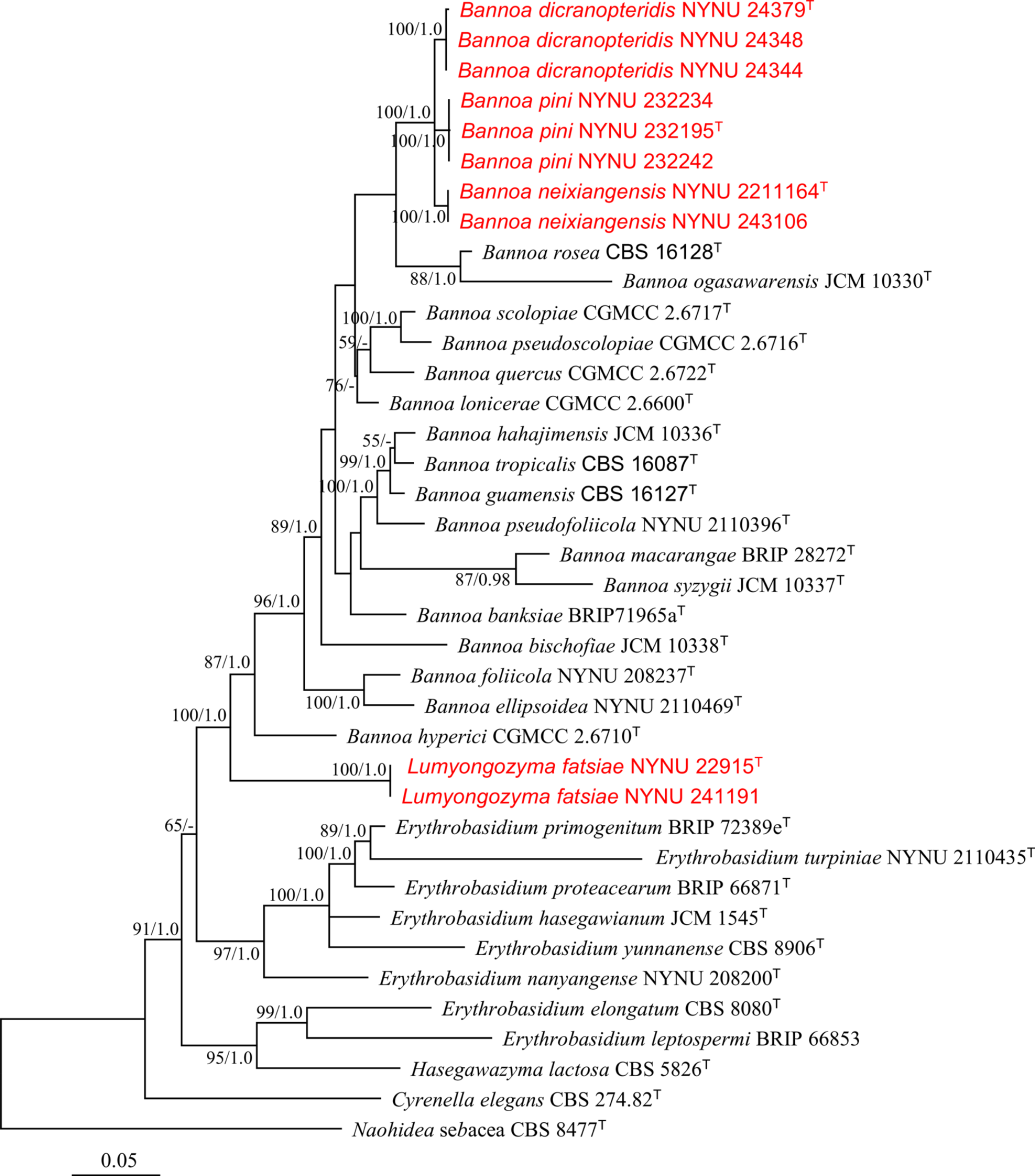
Maximum likelihood (ML) phylogram of Erythrobasidiaceae based on the combined SSU, ITS, LSU, and *TEF1-α* sequence dataset. *Naohidea sebacea* CBS 8477^T^ was used as the outgroup. The bootstrap (BS) values and the Bayesian posterior probabilities (BPPs) above 50% and 0.95 are shown at the nodes. Sequences from type strains are marked with (T), and the new species is indicated in red.

Eight strains, NYNU 232195, NYNU 232234, NYNU 232242, NYNU 2211164, NYNU 243106, NYNU 24379, NYNU 24344, and NYNU 24348, isolated in this study, were separated into three groups within the genus *Bannoa* (Fig. 1). Strains NYNU 232195, NYNU 232234, and NYNU 232242 shared identical LSU sequences and differed by three nucleotides (nt) in the ITS region, indicating they are conspecific. Strains NYNU 2211164 and NYNU 243106 possessed the same LSU and ITS sequences, suggesting they are also conspecific. Group NYNU 2211164 was closely related to group NYNU 232195, differing by four nt substitutions (0.7%) in the LSU region and 9–10 nt mismatches (1.6–1.8%) in the ITS region. Strains NYNU 24379, NYNU 24344, and NYNU 24348 had similar sequences with one nt and three nt difference in the LSU and ITS regions, respectively, indicating they belong to the same species. Group NYNU 24379 clustered with groups NYNU 232195 and NYNU 2211164, but differed from these two groups by 3–4 nt substitutions (0.5–0.7%) in the LSU region and 9–13 nt mismatches (1.6–2.3%) in the ITS region. Based on these sequence comparisons, three novel species of *Bannoa* are proposed: *Bannoa pini*, *B. neixiangensis*, and *B. dicranopteri*.

Two isolates, NYNU 22915 and NYNU 2411221, shared identical LSU and ITS sequences, indicating that they are conspecific. The LSU BLASTn search showed 96% similarity with the genus *Erythrobasidium*. However, further BLASTn searches revealed a lower similarity (91%) in the ITS region with the genus *Bannoa*. Multi-gene phylogenetic analysis placed the two isolates in a basal position within Erythrobasidiaceae. These results prompted us to reconsider whether these strains should be classified in a newly established genus rather than within *Bannoa* or *Erythrobasidium*. As a result, we propose a new genus *Lumyongozyma* and species *L. fatsiae* to accommodate isolates NYNU 22915 and NYNU 2411221.

### Taxonomy

#### Bannoa dicranopteri

Taxon classificationFungiErythrobasidialesErythrobasidiaceae

C.Y. Chai & F.L. Hui sp. nov.

FED88FB7-EEA3-542B-AA12-536340C7B197

861399

Fig. 2

##### Etymology.

The specific epithet dicranopteri refers to *Dicranopteris*, the plant genus from which the type strain was isolated.

**Figure 2.**
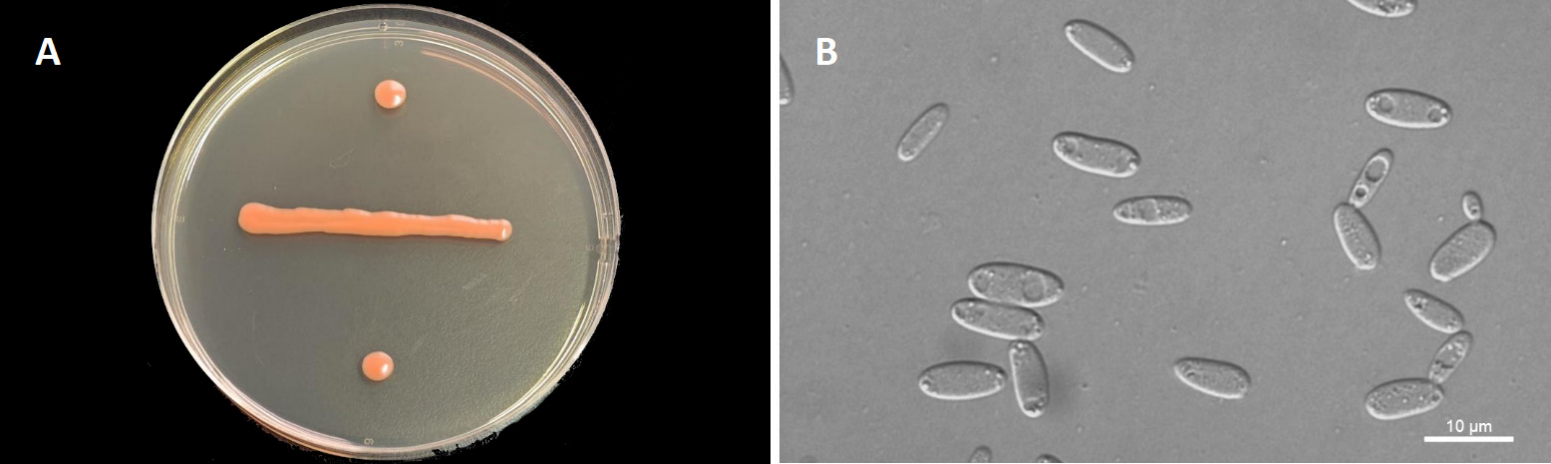
Morphological characteristics of *B. dicranopteri* NYUN 24379. **A**. The streak culture grown on YM agar after 7 d at 20 °C; **B**. Budding cells grown in YM broth for 3 d at 20 °C. Scale bars: 10 μm.

##### Typus.

China • Guizhou Province, Pingtang County, Sifangjing Village, on phylloplane of *Dicranopteris pedata*, March 2024, D. Lu, NYUN 24379 (holotype CICC 33639 preserved as a metabolically inactive state, culture ex-type PYCC 10056).

##### Description.

On YM agar, after 7 d at 20 °C, colonies are orange, smooth, glistening and butyrous in texture. The margin is entire. In YM broth, after 3 d at 20 °C, cells are cylindrical, 2.9–8.1 × 4.8–11.1 μm and single, budding is polar. After 1 mo at 20 °C, a ring and a sediment are present. In Dalmau plate culture on corn meal agar, pseudohyphae are not formed. Sexual structures are not observed on CMA, PDA, and YCBS agar. Ballistoconidia are not produced. Glucose fermentation is absent. Glucose, sucrose, raffinose, melibiose, galactose, trehalose, maltose, melezitose, methyl-α-D-glucoside (weak and delayed), cellobiose, salicin, L-sorbose, L-rhamnose, D-xylose, L-arabinose, D-arabinose (weak and delayed), 5-keto-D-gluconate (weak), glycerol, ribitol (delayed), D-mannitol, D-glucitol, DL-lactate, succinate (weak and delayed), D-gluconate, 2-keto-D-gluconate, D-glucuronate, and D-glucono-1,5-lactone are assimilated as sole carbon. Inulin, lactose, D-ribose, methanol, ethanol, erythritol, galactitol, myo-inositol, citrate, D-glucosamine, and N-acetyl-D-glucosamine are not assimilated. L-Lysine is assimilated as sole nitrogen sources. Nitrate, nitrite, ethylamine, and cadaverine are not assimilated. Maximum growth temperature is 20 °C. Growth on 50% (w/w) glucose-yeast extract agar is negative. No growth in the absence of vitamin-free medium. Starch-like substances are not produced. Urease activity is positive. Diazonium Blue B reaction is positive.

##### Additional strain examined.

China • Guizhou Province, Pingtang County, Sifangjing Village, on phylloplane of *Dicranopteris pedata*, March 2024, D. Lu, NYUN 24344 and NYUN 24348.

##### GenBank accession numbers.

Holotype CICC 33639 (SSU: PX551769, ITS: PP837685, D1/D2: PP849685, *TEF1-α*: PX505481); additional strains NYUN 24344 (SSU: PX551768, ITS: PP837684, D1/D2: PP837681, *TEF1-α*: PX505487) and NYUN 24348 (SSU: PX551770, D1/D2: PP837687, *TEF1-α*: PX505482).

##### Note.

Physiologically, *B. dicranopteri* differs from the closely related species *B. dicranopteri* and *B. neixiangensis* in its ability to assimilate L-lysine and its inability to assimilate inulin and to grow at 25 °C (Table 2).

**Table 2.** Physiological and biochemical characteristics that differ between the new species and closely related species.

Characteristics	*B. dicranopteri*	*B. neixiangensis*	*B. pini*
Carbon assimilation			
Inulin	–	+	w
Lactose	+	+	+
Galactitol	–	–	+
D-Glucono-1,5-lactone	+	–	+
Myo-Inositol	–	–	+
Nitrogen assimilation			
L-Lysine	+	–	–
Growth tests			
Growth at 25 °C	–	+	+

Note. +, positive reaction; –, negative reaction; w, weakly positive.

#### Bannoa neixiangensis

Taxon classificationFungiErythrobasidialesErythrobasidiaceae

C.Y. Chai & F.L. Hui sp. nov.

3297297B-A3BF-58D8-84E8-E07D10A3BF9E

861400

Fig. 3A, B, C

##### Etymology.

The specific epithet neixiangensis refers to the geographic origin of the type strain, Neixiang County, Henan Province.

**Figure 3.**
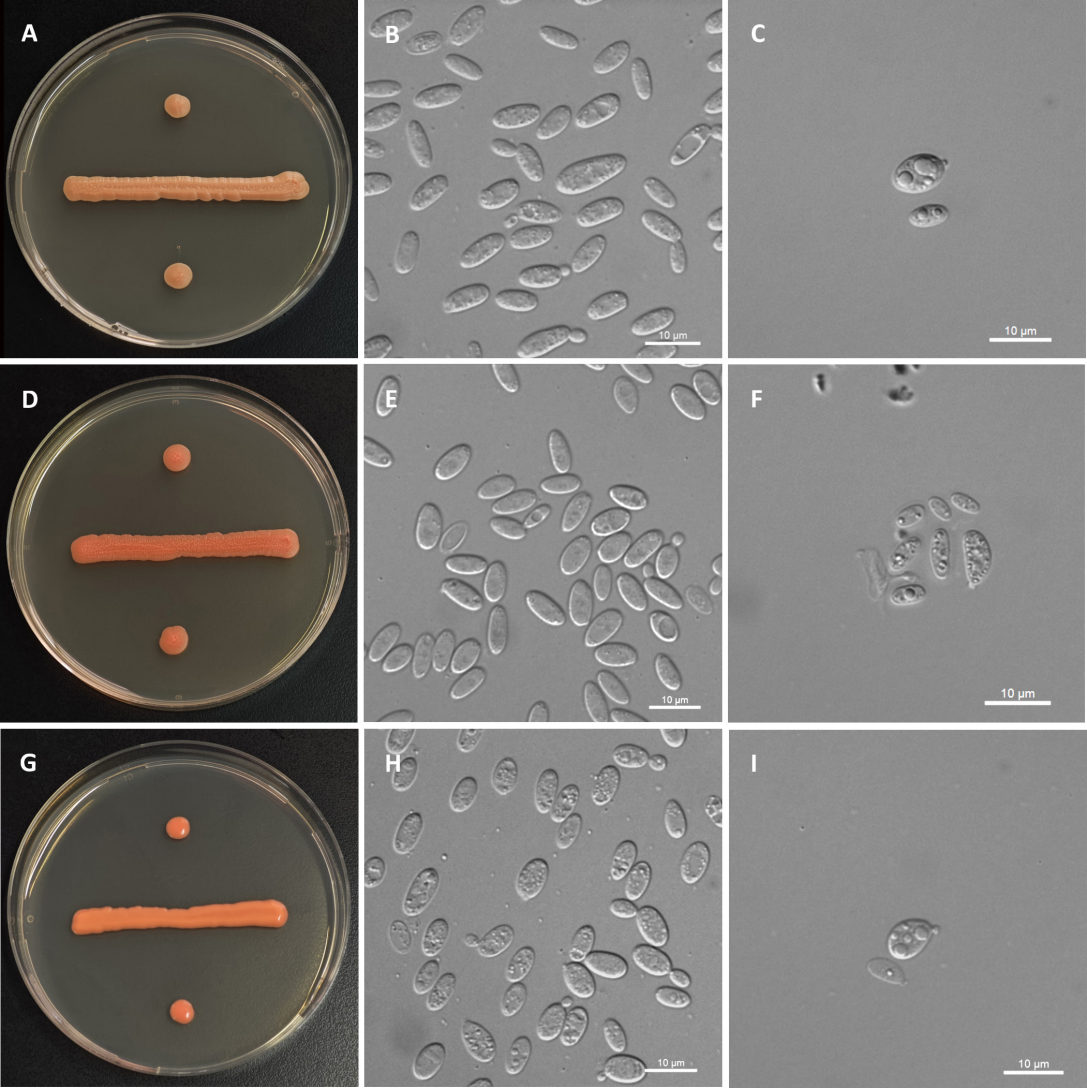
The streak culture grown on YM agar for 7 d at 20 °C, budding cells grown in YM broth for 3 d at 20 °C, and ballistoconidia produced on corn meal agar after 7 d at 17 °C. **A, B, C**. *B. neixiangensis* NYNU 2211164; **D, E, F**. *B. pini* NYNU 232195; **G, H, I**. *L. fatsiae* NYNU 22915. Scale bars: 10 μm.

##### Typus.

China • Henan Province, Neixiang County, Baotianman Nature Reserve, on phylloplane of undetermined leaf, September 2022, J.Z Li, NYNU 2211164 (holotype GDMCC 2.334 preserved as a metabolically inactive state, culture ex-type PYCC 9956).

##### Description.

On YM agar, after 7 d at 20 °C, colonies are orange, smooth, glistening and butyrous in texture. The margin is entire. In YM broth, after 3 d at 20 °C, cells are cylindrical, 3.2–5.5 × 7.0–12.2 μm and single, budding is polar. After 1 mo at 20 °C, a ring and a sediment are present. In Dalmau plate culture on corn meal agar, pseudohyphae are not formed. Sexual structures are not observed on CMA, PDA, and YCBS agar. Ballistoconidia are ovoid and ellipsoidal. Glucose fermentation is absent. Glucose, inulin, sucrose, raffinose, melibiose, galactose, trehalose, maltose, melezitose, methyl-α-D-glucoside (weak and delayed), cellobiose (delayed), salicin, L-sorbose, L-rhamnose, D-xylose, L-arabinose, D-arabinose (weak), 5-keto-D-gluconate (weak), glycerol, ribitol, D-mannitol, D-glucitol, DL-lactate (weak and delayed), succinate (delayed), D-gluconate (weak), 2-keto-D-gluconate (weak and delayed), and D-glucuronate (delayed) are assimilated as sole carbon. Lactose, D-ribose, methanol, ethanol, erythritol, galactitol, myo-inositol, citrate, D-glucosamine, N-acetyl-D-glucosamine, and D-glucono-1,5-lactone are not assimilated. Nitrate, nitrite, ethylamine, L-lysine, and cadaverine are not assimilated. Maximum growth temperature is 25 °C. Growth on 50% (w/w) glucose-yeast extract agar is negative. No growth in the absence of vitamin-free medium. Starch-like substances are not produced. Urease activity is positive. Diazonium Blue B reaction is positive.

##### Additional strain examined.

China • Guizhou Province, Pingtang County, Sifangjing Village, on phylloplane of *0rmosia henryi*, March 2024, D. Lu, NYNU 243106.

##### GenBank accession numbers.

Holotype GDMCC 2.334 (SSU: PX551774, ITS: OP954739, D1/D2: OP954732, *TEF1-α*: OP750517); additional strain NYNU 243106 (SSU: PX551775, ITS: PX551776, D1/D2: PX551777, *TEF1-α*: PX505486).

##### Note.

Physiologically, *B. neixiangensis* difers from the closely related species *B. pini* in its inability to assimilate galactitol, D-glucono-1,5-lactone, and myo-inositol (Table 2).

#### Bannoa pini

Taxon classificationFungiErythrobasidialesErythrobasidiaceae

C.Y. Chai & F.L. Hui sp. nov.

B426854E-C63B-5E37-941E-00FBF1B9E8B0

861401

Fig. 3D, E, F

##### Etymology.

The specific epithet pini refers to *Pinus*, the plant genus from which the type strain was isolated.

##### Typus.

China • Guizhou Province, Pingtang County, Sifangjing Village, on phylloplane of *Pinus massoniana*, February 2023, D. Lu, NYNU 232195 (holotype GDMCC 2.496 preserved as a metabolically inactive state, culture ex-type PYCC 9981).

##### Description.

On YM agar, after 7 d at 20 °C, colonies are dark pink, smooth, glistening and butyrous in texture. The margin is entire. In YM broth, after 3 d at 20 °C, cells are ellipsoidal and cylindrical, 3.6–5.1 × 7.2–9.0 μm and single, budding is polar. After 1 mo at 20 °C, a ring and a sediment are present. In Dalmau plate culture on corn meal agar, pseudohyphae are not formed. Sexual structures are not observed on CMA, PDA, and YCBS agar. Ballistoconidia are falcate or cylindrical. Glucose fermentation is absent. Glucose, inulin (weak), sucrose, raffinose, melibiose, galactose, lactose (delayed), trehalose, maltose, melezitose, methyl-α-D-glucoside (delayed), cellobiose, salicin, L-sorbose, L-rhamnose, D-xylose, L-arabinose, D-arabinose, 5-keto-D-gluconate, D-ribose, glycerol, ribitol, galactitol, D-mannitol, D-glucitol, myo-inositol, DL-lactate (delayed), succinate, D-gluconate, 2-keto-D-gluconate, D-glucuronate, and D-glucono-1,5-lactone are assimilated as sole carbon. Methanol, ethanol, erythritol, citrate, D-glucosamine, and N-acetyl-D-glucosamine are not assimilated. L-Lysine is assimilated as sole nitrogen sources. Nitrate, nitrite, ethylamine, and cadaverine are not assimilated. Maximum growth temperature is 25 °C. Growth on 50% (w/w) glucose-yeast extract agar is negative. No growth in the absence of vitamin-free medium. Starch-like substances are not produced. Urease activity is positive. Diazonium Blue B reaction is positive.

##### Additional strain examined.

China • Guizhou Province, Pingtang County, Sifangjing Village, on phylloplane of *Cunninghamia lanceolata*, February 2023, D. Lu, NYUN 232234; China • Guizhou Province, Pingtang County, Sifangjing Village, in phylloplane of *Distylium racemosum*, February 2023, D. Lu, NYUN 232242

##### GenBank accession numbers.

Holotype GDMCC 2.496 (SSU: PX551771, ITS: OQ851908, D1/D2: OQ851907, *TEF1-α*: PX505484); additional strains NYUN 232234 (SSU: PX551772, ITS: OQ851909, D1/D2: OQ857289) and NYUN 232242 (SSU: PX551773, ITS: OQ857287, D1/D2: OQ857288, *TEF1-α*: PX505485).

##### Note.

Physiologically, *B. pini* difers from the closely related species *B. neixiangensis* in its ability to assimilate galactitol, D-glucono-1,5-lactone, and myo-inositol (Table 2).

#### Lumyongozyma

Taxon classificationFungi

C.Y. Chai & F.L. Hui gen. nov.

699FB39F-87DA-5D41-9FB4-FD022C63719F

861402

##### Type species.

*Lumyongozyma fatsiae*

##### Etymology.

The genus is named in honor of Saisamorn Lumyong for her contributions to yeast taxonomy.

##### Description.

The genus can be distinguished from the closely related genus *Bannoa* based on phylogenetic analyses and phenotypic characteristics. Colonies are dark pink and butyrous in texture. Budding cells are present, and yeast cells divide by polar budding. Pseudohyphae and hyphae are not produced. Sexual structures are not observed in either individual or mixed cultures. Glucose fermentation does not occur, and starch-like substances are not produced. The diazonium blue B reaction and urease activity are both positive.

##### Classification.

Erythrobasidiaceae, Erythrobasidiales, Cystobasidiomycetes, Pucciniomycotina, Basidiomycota.

#### Lumyongozyma fatsiae

Taxon classificationFungiErythrobasidialesErythrobasidiaceae

C.Y. Chai & F.L. Hui sp. nov.

2431019B-DC67-5B44-8507-DB970281863A

861403

Fig. 3G, H, I

##### Etymology.

The specific epithet fatsiae refers to *Fatsia*, the plant genus from which the type strain was isolated.

##### Typus.

China • Guizhou Province, Guiyang City, Guiyang Botanical Garden, on phylloplane of *Fatsia japonica*, August 2022, L. Zhang, NYNU 22915 (holotype GDMCC 2.296 preserved as a metabolically inactive state, culture ex-type PYCC 9936).

##### Description.

On YM agar, after 7 d at 20 °C, colonies are dark pink, smooth, glistening and butyrous in texture. The margin is entire. In YM broth, after 3 d at 20 °C, cells are ellipsoidal and cylindrical, 3.4–4.8 × 4.8–7.9 μm and single, budding is polar. After 1 mo at 20 °C, a pellicle and a sediment are present. In Dalmau plate culture on corn meal agar, pseudohyphae are not formed. Sexual structures are not observed on CMA, PDA, and YCBS agar. Ballistoconidia are allantoid or reniform. Glucose fermentation is absent. Glucose, sucrose, raffinose, melibiose, galactose, lactose (delayed), trehalose, maltose, melezitose, methyl-α-D-glucoside, cellobiose, L-sorbose (delayed), D-xylose, D-mannitol, D-glucitol, myo-inositol, D-gluconate, 2-keto-D-gluconate, and D-glucuronate (weak and delayed) are assimilated as sole carbon. Inulin, salicin, L-rhamnose, L-arabinose, D-arabinose, 5-keto-D-gluconate, D-ribose, methanol, ethanol, glycerol, erythritol, ribitol, galactitol, DL-lactate, succinate, citrate, D-glucosamine, N-acetyl-D-glucosamine, and D-glucono-1,5-lactone are not assimilated. L-Lysine is assimilated as sole nitrogen sources. Nitrate, nitrite, ethylamine, and cadaverine are not assimilated. Maximum growth temperature is 30 °C. Growth on 50% (w/w) glucose-yeast extract agar is negative. No growth in the absence of vitamin-free medium. Starch-like substances are not produced. Urease activity is positive. Diazonium Blue B reaction is positive.

##### Additional strain examined.

China • Guizhou Province, Guiyang City, Guiyang Botanical Garden, on phylloplane of *Jasminum mesnyi*, October 2024, D. Lu, NYUN 241191.

##### GenBank accession numbers.

Holotype GDMCC 2.296 (SSU: PX444847, ITS: OP740374, D1/D2: OP740373, *TEF1-α*: PX505479); additional strain NYUN 241191 (SSU: PX551767, ITS: PX551843, D1/D2: PX551778, TEF1-α: PX505480).

## Discussion

In this study, one new genus, *Lumyongozyma*, typified by the new species *L. fatsiae*, along with three novel species of *Bannoa*: *B. dicranopteri*, *B. neixiangensis*, and *B. pini*, were described based on phylogenetic and morphological evidence. These newly recognized species are distributed across different regions of China. Future studies will likely uncover more species of this genus and related genera, contributing new evidence and facilitating a deeper understanding of the relationships within and among these taxa.

The genus *Bannoa* is known as a representative group of ballistosporous yeasts. Most species of this genus typically produce ballistoconidia, which can be observed as an opaque mirror image on the lid of an inverted Petri dish, formed by the discharged spores (do Carmo-Sousa and Phaff 1962; Kurtzman et al. 2011). However, the production of ballistoconidia is influenced by cultivation methods and can vary between clones (Nakase et al. 1993; Nakase 2000). In this study, the two strains of the new species *B. dicranopteri* were identified as non-ballistoconidium-forming yeasts. This phenomenon is extremely rare in the genus *Bannoa*. The presence of this species may be the result of yeasts produced by vegetative cells inhabiting the phylloplane.

Members of *Bannoa* are rarely isolated in pure culture. This is likely due to the commonly used “ballistospore-fall method”. If other methods, such as leaf imprinting, dilution plating, or enrichment isolation are used, this may increase the chances of detecting this genus (and possibly others) from leaves. Other factors, including types of media and incubation temperature, may also affect isolation results (Nakase et al. 2001; Limtong and Koowadjanakul 2012). Currently, 17 species are recognized in the genus (Sun et al. 2025). Among these, *B. hahajimensis* and *B. tropicalis* are known from their filamentous morphs (Hamamoto et al. 2002; Parra and Aime 2019). The other 15 species are known only in their yeast morph and reproduce by polar budding (Hamamoto et al. 2002; Parra and Aime 2019; Chai et al. 2023; Sun et al. 2025). In this study, we introduce three new species of *Bannoa*: *B. dicranopteri*, *B. neixiangensis*, and *B. pini*, and describe them in their yeast morphs. Based on cultivation experiments by Schoutteten et al. (2024) and comparisons with other yeast-dominated classes such as Microbotryomycetes, it is highly likely that most, if not all, *Bannoa* species are dimorphic fungi, with the filamentous morph still to be discovered (Schoutteten et al. 2023). Since the filamentous morph is associated with basidia production and sexual reproduction, crossing experiments between compatible yeast strains may lead to the discovery of filamentous morphs under laboratory conditions for species currently known only as yeasts. Further cultivation efforts may facilitate the isolation of these missing filamentous morphs of *Bannoa* in pure culture or through co-cultivation experiments. Establishing the connection between yeast and filamentous stages will significantly enhance our understanding of the genus *Bannoa*.

*Erythrobasidium*, the type genus of the family Erythrobasidiaceae, was introduced by Hamamoto et al. (1988) to accommodate *E. hasegawae*, the teleomorphic state of *Rhodotorula hasegawae*. Currently, eight species are recognized in the genus (Tan and Shivas 2024; Lu et al. 2024). Previous studies indicated that *E. elongatum* and *E. leptospermi* formed two long branches at the base of the *Erythrobasidium* clade with low support (Tan and Shivas 2022; Lu et al. 2024; Tan and Shivas 2024). However, our phylogenetic analysis revealed that these two species are not closely related to the *Erythrobasidium* lineage containing the type species, but instead form a separate branch basal to the *Hasegawazyma lactosa* lineage, with high support. Therefore, further analyses using additional molecular or genomic data are required to clarify their phylogenetic positions.

## Conclusion

The current study introduces one new monotypic genus, *Lumyongozyma*, typified by the new species *L. fatsiae*, along with three new species of *Bannoa*: *B. dicranopteri*, *B. neixiangensis*, and *B. pini*, all isolated from leaf surfaces in different regions of China. Phylogenetic analysis and phenotypic characterization support the classification of these four new taxa within Erythrobasidiaceae (Fig. 1). Detailed descriptions, illustrations, and comparative discussions with their closest relatives are provided. Our study highlights the novel geographical distribution and high species diversity of *Bannoa* in tropical and subtropical China. These findings contribute to the understanding of generic and species diversity within this family. Moving forward, we anticipate the discovery of more species and genera within this family.

## Supplementary Material

XML Treatment for Bannoa dicranopteri

XML Treatment for Bannoa neixiangensis

XML Treatment for Bannoa pini

XML Treatment for Lumyongozyma

XML Treatment for Lumyongozyma fatsiae

## Additional information

### Use of AI

No use of AI was reported.

### Author ORCIDs

Zhi-Wen Xi https://orcid.org/0000-0002-5814-5283

Bing-Yan Song https://orcid.org/0009-0000-6802-5077

Chun-Yue Chai https://orcid.org/0000-0001-7753-6223

Qiu-Hong Niu https://orcid.org/0000-0003-1695-7117

Feng-Li Hui https://orcid.org/0000-0001-7928-3055

### Data availability

All gene sequences generated in this study are available in GenBank under the accession numbers provided. All of the data that support the findings of this study are available in the main text.
